# The atrial fibrillation epidemic is approaching the physician’s door: will mobile technology improve detection?

**DOI:** 10.1186/s12916-014-0180-8

**Published:** 2014-09-29

**Authors:** Perttu J Lindsberg, Lauri Toivonen, Hans-Christoph Diener

**Affiliations:** Department of Neurology, Helsinki University Central Hospital, P.O. Box 700, Helsinki, Finland; Research Programs Unit, Molecular Neurology, Biomedicum Helsinki, and Department of Clinical Neurosciences, University of Helsinki, P.O. Box 700, Helsinki, Finland; Cardiac Electrophysiology, Heart and Lung Center, Helsinki University Central Hospital, Haartmaninkatu 4, 00290 Helsinki, Finland; Department of Neurology, University Hospital Essen, Hufelandstrasse 55, Essen, 45147 Germany

**Keywords:** Atrial fibrillation, Arrhythmia, Cardiac monitoring, Embolic stroke, Disease screening, Telemedicine, Personal medical application

## Abstract

The rising numbers of people with atrial fibrillation (AF) carry a heavy toll on our graying population. Epidemiological data suggest that AF exists in 1 in 10 individuals aged older than 80 years. The risk of embolic stroke increases along with well-known cardiovascular risk factors. Should there be systematic screening for the elderly? Although 1 in 10 is a huge hit rate in screening for any major illness, the initiative for such programs in AF remains in ‘research and development’.

At present, cardiologists can utilize implantable loop recorders in patients referred for specialist consultation. Novel technologies are also available, including cloud-based, algorithm-assisted, non-invasive monitoring patches, which allow extended observation periods.

What about people in the community without a recognized need for cardiologic investigation? Mobile technology has made detection of pulse irregularity possible without medical attention. Smartphone apps enable opportunistic rhythm monitoring, but true arrhythmias need to be medically verified. AF may be the first common disorder to be effectively screened for by mobile technology. In the spirit of proactive campaigns such as ‘*Know Your Pulse’*, we should prepare for rapidly increasing reports of various pulse irregularities.

The purpose of this article is to promote more vigorous detection of undetected, quiet persistent or paroxysmal AF. Lack of efficient screening and absence of versatile AF detection technology comprise an important bottleneck in the prevention of first strokes. We believe that more aggressive societal programs and technological innovations in self-administered AF detection could lead to a reduced burden of embolic strokes in the face of the AF epidemic related to the ‘graying’ of western civilization.

## Criteria for systematic AF screening

AF fulfills key premises for a condition to be systematically screened for in the healthy population [1,2]. We comment on a few of these in Table 1.

**Table 1:** **Summary of key premises for a condition to be systematically screened for in the healthy population**

**Criteria for systematic AF screening** ^**1.2**^	
AF and stroke as societal health challenges	
1)	AF is common, affecting approximately 5% of adults aged 65 years and older, and 10% of those older than 80 years [3]. One in four individuals is now being projected to develop the condition in their lifetime [4]. Investigators of one community survey reported a rise of 12.6% in the incidence of AF during the past 2 decades, and projected that 15.9 million people in the USA will have the disorder by 2050 [5]
2)	AF increases the risk for ischemic stroke by approximately fivefold. In the UK, findings from the SAFE study [6] showed a baseline prevalence of AF of 7.2% in patients aged 65 years and older, with an increased prevalence in men (7.8%) and in those aged 75 years and older (10.3%), and a yearly incidence of new AF of about 1.6%. AF causes 15% of all strokes, and 30% of those whose strokes occur after the age of 80 years in the US population [3,4]
3)	AF constitutes a public health burden by triggering prevalent embolic strokes, and it frequently leads to impaired quality of life, resulting in high healthcare costs. The cost of stroke is substantial. In the UK, mean censor-adjusted 5-year hospital costs after stroke were $25,741 [7], but the lifetime costs have been estimated to be substantially higher; for example, $130,000 after ischemic stroke in Finland [8]
Possibilities for AF screening	
4)	Individuals of advanced age – for example, 70 and 80 years old –are a suitable target population for screening, as the incidence of AF at older ages is substantial
5)	There is an early or latent stage, in which patients with AF are commonly asymptomatic, but they may progress to manifest cardiac problems and sudden cardioembolism and stroke. Further, AF may be preceded by subclinical atrial tachyarrhythmias, which are associated with significantly increased risk of stroke and systemic embolism [9]
6)	The diagnosis is simple using widely available, non-invasive tools such as ECG and Holter-ECG
Recommendations for treatment	
7)	There are already agreed policies in place for treatment of incidental AF in asymptomatic individuals, or in patients following symptoms or stroke. Several accepted interventions are available, which can correct the underlying cardiac rhythm disturbance of AF, including pharmacologic cardioversion, electrical cardioversion, and catheter ablation [10]
8)	Scoring systems (for example, the CHA_2_DS_2_-VASc score) detailed in consensus guidelines are in place to individually guide the initiation of oral OACs in order to decrease the risk of subsequent AF-related new ischemic strokes [11]. OACs produce marked reductions in strokes; for example, up to 64% for warfarin and an estimated 77% for dabigatran compared with placebo [11]
9)	Several novel OACs, such as dabigatran, rivaroxaban and apixaban, are available, which probably reduces the threshold of initiating long-term therapy to reduce cardioembolic strokes [12-15].
Risks and benefits	
10)	Potential risks of screening include development of serious hemorrhagic complications in some patients prescribed OACs.
11)	Scientific evidence for the benefits and effectiveness of screening programs is still being produced. It has not yet been demonstrated that systematic screening for AF improves outcome

*AF* atrial fibrillation, *ECG* electrocardiogram, *OAC* oral anti-coagulant, *SAFE* Screening for Atrial Fibrillation in the Elderly.

## Modes and methods of AF detection

Methods for initial detection of AF are dependent on the presence or absence of symptoms. In asymptomatic subjects, pulse palpation and interpretation of incidental 12-lead or bipolar ECGs are the most frequently used methods. One study found that pulse palpation by a practice nurse plus 12-lead ECG reading by a general practitioner is an efficient means of screening older patients for AF [16]. However, inviting elderly patients with or without risk factors to attend for a 12-lead ECG is not cost-effective, as it produces small benefit at the cost of high workload. Instead, campaigns promoting regular pulse palpation, such as *Know Your Pulse*, have been devised.

In symptomatic patients and in those suspected of having paroxysmal AF or cryptogenic stroke, AF is often detected and confirmed by serial ECGs or Holter monitoring for 1 or 2 days. In those with infrequent paroxysms, long-term continuous ECG by automatic (for asymptomatic patients) or patient-activated symptomatic event loop recorders might be needed. Quantification of arrhythmia burden with implantable devices (for example, implantable loop recorder; ILR) may be used in attempt to provide an individual estimate of AF burden and related stroke risk. In some patients with existing cardiac arrhythmia, recorded or remote surveillance of implanted cardiac arrhythmia devices may provide a diagnosis. Newer methods include modified sphygmomanometers or finger-probe devices.

## Why is systematic screening for AF not carried out in at-risk populations?

AF not only increases the risk for ischemic stroke by approximately fivefold, but AF-related strokes result in more deaths and disability than strokes from other causes [17]. Despite this, the US Preventive Services Task Force and the UK National Screening Committee have no policy recommendation for systematic or opportunistic (for example, pulse recording with follow-up ECG) screening for AF in primary care or in the healthy population.

Detection of AF causes discomfort for otherwise healthy individuals and apparent added direct healthcare costs for society. However, societal reasons for not performing more systematic screening of AF are offset by the decreased need for regular blood coagulation tests with newer oral anti-coagulants (OACs), which have been demonstrated as cost-effective [18].

## Risk–benefit ratio of systematic AF screening

Could enhanced AF screening produce more harm than benefit? The elevated stroke risk in AF is not homogeneous, and changes cumulatively with the presence of stroke risk factors, as illustrated in various stroke risk stratification schema such as CHADS_2_ score and the newer CHA_2_DS_2_-VASc score [11]. These categorize patients as having low, moderate, or high risk. Management guidelines have traditionally recommended that patients with high or moderate risk should be given oral anti-coagulation, whereas the current guidelines recommend oral anti-coagulation for patients with intermediary risk of CHA_2_DS_2_-VASc score 1 or 2 as well. Anti-platelet agents can be given when oral anti-coagulation therapy cannot be given [19].

However, this approach also requires consideration of bleeding risk. To this end, the HAS-BLED score (uncontrolled Hypertension, Abnormal renal/liver function, Stroke, Bleeding history or predisposition, Labile international normalized ratio, Elderly >65 years, Drugs/alcohol concomitantly) has been proposed as a simple bleeding-risk assessment for patients with AF [11]. Standardized assessment of bleeding risk allows informed decision-making, and alerts clinicians to potentially correctable bleeding risk factors. We believe that by applying these schemes to estimate the individual risk/harm profile, it is possible to balance the risks and benefits in a given patient in order to reduce their stroke risk. Screening should always be accompanied by improved patient management.

We are aware of the perennial challenges relating to screening policy decisions. The exact fraction of strokes caused by undetected AFs versus those already detected cannot be predicted. Given the high prevalence of AF in the elderly population, it is unlikely that opportunistic AF screening by pulse palpation during occasional primary care visits will essentially change the numbers of detected AF in the target population. Campaigns such as ‘*Know Your Pulse*’ are important endeavors for educating the general public, although data on their efficacy are lacking. One solution would be to have nurses in doctors’ offices routinely take the pulse of people aged over 70 years, who see a doctor several times a year on average.

We are not convinced that the projected yield of palpation campaigns will be satisfactory in tackling the AF epidemic. Therefore, given the advent of novel technological advances, we encourage additional steps. To this end, the yield from systematic population screening for AF in healthy individuals with normal ECG was recently investigated. All inhabitants in the municipality of Halmstad (Sweden) aged 75 to 76 years were invited to a stepwise screening program for AF [20]. In the first step, participants underwent 12-lead ECG, and reported their relevant medical history. Those with sinus rhythm on 12-lead ECG, no history of AF, and two or more risk factors according to CHADS_2_ score, were invited to participate in a 2-week recording period using a hand-held ECG, and asked to record 20 or 30 seconds of ECG twice daily and if palpitations occurred. In total, 1,330 inhabitants were invited, of whom 848 (64%) participated. Previously undiagnosed silent AF was found in 10 (1%) of 848 individuals who recorded 12-lead ECG. Of 81 patients with known AF, 35 (43%) were not on OAC treatment. Of 403 individuals with two or more risk factors for stroke who completed the hand-held ECG event recording, 30 (7.4%) were diagnosed with paroxysmal AF. Thus, 75/848 (9%) of the screened population were candidates for new OAC treatment, of whom 57 actually started this treatment [20]. An approach for future research would be to utilize known biomarkers of AF to complement CHA_2_DS_2_-VASc scores in selecting individuals for AF screening.

## Remote surveillance technology for AF detection

Stroke researchers are currently attempting to improve rates of AF detection in cryptogenic stroke with continuous automated ECG analysis at the bedside in stroke units or with extended recordings using implantable cardiac monitoring devices [21]. However, we argue that given the high prevalence of AF, physicians in general should promote detection of silent AF in individuals well before the potentially fatal or debilitating ‘index’ stroke, that is, for primary prevention in the general population. In particular, elderly individuals are at considerable risk of having undetected paroxysmal or persistent AF.

Despite ILRs having been available for nearly a decade, their use for AF screening has not become common. The IMPACT Study was designed to investigate the clinical benefit of combined use of home monitoring technology and a predefined anti-coagulation plan compared with conventional device evaluation and physician-directed anti-coagulation in patients with implanted dual-chamber defibrillators or cardiac resynchronization therapy devices [22]. The event rates were presumably low, as this trial was terminated prematurely. The trial included only patients with existing cardiac indication and CHADS_2_ score of 1 or greater, and had no special focus on elderly individuals. In addition, the control subjects were patients monitored in accordance with guidelines for management of patients with implanted cardiac arrhythmia devices, in whom the rates of thromboembolic events are relatively low. Therefore, studies on elevated cardiovascular and thromboembolic risk in a broader population of advanced age might be better for investigating differential efficacies in AF detection. Secular trends transform the settings and technologies by which pulse irregularity and newly diagnosed AF are initially detected. More AF cases are detected outside hospitals, and more pulse irregularities are probably initially detected without ECG in asymptomatic subjects.

We postulate that AF will soon be the first major cardiovascular disease to be detected by personal devices that are rapidly becoming available worldwide. Currently, lightweight insertable cardiac monitors are being manufactured for capturing ECG for collapse diagnostics. Computer-assisted decision support software used in the clinic to detect AF has yielded a specificity of greater than 99% and sensitivity of 87% [6]. Afib Alert® (Lohman Technologies, Sussex, WI, USA) is a Food and Drug Administration (FDA)-approved device that helps users know when they are likely to be experiencing AF. It allows a patient using the device to communicate information to their doctor directly over their phone. Another application is the ZIO® Patch (iRhythm Technologies, San Francisco, CA, USA), an algorithm-assisted device allowing cloud-based AF detection monitoring for an extended period [23]. A doctor's prescription is generally required for these devices; however, it is unlikely that this will prevent comparable devices from eventually being distributed to the general public by manufacturers and retailers for considerable profits.

## Self-administered irregular pulse detection: solution or new concern?

For systematic screening to be demonstrated to be an effective intervention, it must improve the detection rate of AF and provide benefit for those in whom AF is detected early. This has yet to be demonstrated [24]. In view of the high cost of systematic screening programs, it may turn out that cost-effectiveness is difficult to attain. Furthermore, absence of AF at one screening time-point does not preclude its later development, thus techniques usable at any time according to the patient’s initiative will be more useful, especially in high-risk individuals.

This story is likely to take an unexpected turn and start outside the healthcare system. Pulse-detection technology is readily available in all sports retailers, and may soon be refined to detect an irregular pulse as well. Pulse detection is also being built into smartphones and wrist watches, which is likely to expand personal rhythm detection sooner than we think. Already, there are smartphone apps that recognize an irregular pulse without any external devices such as pulse sensors. Illumination of the pulp of one finger placed on the camera lens allows the light sensor of the camera and the application program to detect pulsating color changes and reveal an irregular rhythm in 1 minute. A recent algorithm demonstrated excellent sensitivity (96.2%), specificity (97.5%), and accuracy (96.8%) for beat-to-beat discrimination of an irregular pulse during AF from sinus rhythm [25]. Similar systems have already received FDA approval [26]. However caution is essential, as recent technology with pulse sensoring algorithms within a smartphone or a mobile device is less accurate and less trustworthy than a multiple-lead ECG examination, because irregular inter-beat (RR) intervals do not always indicate AF. False-positive readings may lead to unnecessary false alarms in healthy subjects.

Technological innovations and mobile solutions for irregular pulse detection are likely to bypass efforts to rigorously validate the net benefit of systematic screening campaigns to detect AF. The use of smartphones by elderly individuals is increasing, and these people are aware of the age-related risk of AF and its dangers. Most likely, there will be development of lightweight devices connected to smartphones that will be able to monitor heart rhythm for extended periods of time, such as during exercise and sleep. Any efforts to restrict this self-detection activity to physician prescription only will be futile. These efforts would in fact be counterintuitive, as we already encourage the elderly population to “Know your pulse” using manual palpation. Indeed, we should instead encourage this progress, and start preparing the healthcare system for an increasing number of self-detected cases of AF following self-administered pulse detection, with or without any symptoms.

Doctors will need to answer an escalating number of questions, such as:

- How can I ascertain whether the irregular rhythm that was detected by my device is dangerous?
- What is the critical duration of AF periods at which there is a risk for embolic stroke?
- My device is detecting AF more than once every month. Should I start blood-thinning medication, and is aspirin sufficient when I have no cardiac disease?

To answer these questions, we recommend that studies should be carried out immediately. Evidence-based data are needed on the later clinical course of AF once found by *ad hoc* screening, and even randomized studies could be envisaged to assess therapy protocols to influence the risk of emboli after very short and infrequent episodes of AF.

## Conclusion

AF detection should move forward on two fronts in which modern technology is utilized: 1) implementation of systematic screening of individuals who are healthy but have risk factors for stroke, and 2) innovations in self-administered AF or irregular pulse detection by personal devices. It is time to prepare for an escalating number of suspected AF cases in primary care. It has long been suggested that AF is becoming an epidemic. These developments will soon take the AF epidemic to the physician’s door. This is a welcome development for patients, physicians, and healthcare systems.
